# Formulation and Evaluation of Solid Self-Nanoemulsifying Drug Delivery System of Cannabidiol for Enhanced Solubility and Bioavailability

**DOI:** 10.3390/pharmaceutics17030340

**Published:** 2025-03-06

**Authors:** Fengying Wu, Qing Ma, Guanghui Tian, Kaixian Chen, Rulei Yang, Jingshan Shen

**Affiliations:** 1 School of Chinese Materia Medica, Nanjing University of Chinese Medicine, Nanjing 210023, China; wufengying@simm.ac.cn (F.W.); kxchen@simm.ac.cn (K.C.); 2 State Key Laboratory of Drug Research, Shanghai Institute of Materia Medica, Chinese Academy of Sciences, Shanghai 201203, China; 3 Vigonvita Life Sciences Co., Ltd., Suzhou 215125, China; qing.ma@vigonvita.cn (Q.M.); guanghui.tian@vigonvita.cn (G.T.); 4 University of Chinese Academy of Sciences, Beijing 100049, China

**Keywords:** cannabidiol, SNEDDS, solid SNEDDS, solubility, pharmacokinetics, bioavailability

## Abstract

**Background/Objectives:** This study aims to develop a solid self-nanoemulsifying drug delivery system (SNEDDS) to enhance the solubility and oral bioavailability of cannabidiol (CBD). **Methods:** According to the solubility of CBD and pseudo-ternary phase diagrams of the different ingredients, an oil (medium-chain triglyceride, MCT), mixed surfactants (Labrasol, Tween 80), and a co-surfactant (Transcutol) were selected for the SNEDDS. CBD-loaded SNEDDS formulations were prepared and characterized. The optimal SNEDDS was converted into solid SNEDDS powders via solid carrier adsorption and spray drying techniques. Various evaluations including flowability, drug release, self-emulsifying capacity, X-ray diffraction (XRD), differential scanning calorimetry (DSC), Fourier transform infrared spectroscopy (FTIR), morphology, and pharmacokinetic characteristics were conducted. Subsequently, the solid powders with fillers, disintegrants, and lubricants were added to the capsules for accelerated stability testing. **Results:** The investigations showed that the two S-SNEDDS formulations improved the CBD’s solubility and in vitro drug release, with good storage stability. The pharmacokinetic data of Sprague Dawley rats indicated that a single oral dose of L-SNEDDS and spray drying SNEDDS led to a quicker absorption and a higher Cmax of CBD compared to the two oil-based controls (CBD-sesame oil (similar to Epidiolex^®^) and CBD-MCT), which is favorable for the application of CBD products. **Conclusions:** SNEDDS is a prospective strategy for enhancing the solubility and oral bioavailability of CBD, and solid SNEDDS offers flexibility for developing more CBD-loaded solid formulations. Moreover, SNEDDS provides new concepts and methods for other poorly water-soluble drugs.

## 1. Introduction

*Cannabis sativa* L., being one of the earliest plants cultivated by humans, possesses significant agricultural and medicinal values [1,2]. Cannabidiol (CBD) is the primary non-psychoactive ingredient extracted from cannabis, without hallucinogenic and addictive properties [3,4]. CBD is a promising drug molecule with a variety of pharmacological activities. Recently, the CBD oral solution (Epidiolex^®^, a sesame oil-based formulation) was approved by the US FDA for the treatment of seizures related to the Lennox–Gastaut syndrome, the Dravet syndrome, or the tuberous sclerosis complex in patients who are 1 year of age and older [5]. Although the approved indication for the use of CBD in medicine is to treat refractory epilepsy, a number of clinical studies and reviews have shown that this phytocannabinoid has other various effects, such as anti-anxiety [6,7], anti-psychotic [8,9], sedative, and hypnotic [10] effects, and can be used in the treatment of autism spectrum disorders [11,12], which greatly broadens its therapeutic potential [13]. However, as a typical drug of the Biopharmaceutics Classification System (BCS) Class II [14], CBD has properties such as low aqueous solubility (12.6 mg/L) and high lipophilicity (logP 6.3) [15]. These physiological characteristics of CBD lead to a relatively low oral bioavailability (approximately 9% to 13%) [16], significant first-pass liver metabolism, and variable pharmacokinetic profiles, further restricting its clinical applications [17].

To overcome these limitations, numerous nanotechnology-based formulation approaches have been employed for the efficient delivery of CBD, such as the polymeric nanoparticle [18], lipid nanocapsule (LNC) [19], nanostructured lipid carrier (NLC) [20], and self-nanoemulsifying drug delivery systems (SNEDDSs) [21,22,23,24]. Among these delivery vectors, SNEDDS is primarily utilized to enhance the solubility and absorption of CBD.

SNEDDSs are anhydrous isotropic liquid mixtures (also known as pre-concentrates), constituted by lipophilic drugs combined with lipids, surfactants, and co-surfactants. When exposed to the fluids in the gastrointestinal tract, these systems spontaneously carry out in situ emulsification and form transparent O/W nanoemulsions. The spontaneous emulsification process eventually reduces particle size (less than 200 nm) and maximizes the surface area. The SNEDDS can increase the solubility of drugs with poor water solubility and show outstanding drug loading capacity. SNEDDSs, as stable colloidal delivery systems, can improve solubility, enhance bioavailability, reduce the first-pass metabolism (increase lymphatic absorption), and decrease the individual variation coefficient of CBD [17,25].

Over the years, numerous SNEDDS formulations have been carried out regarding the delivery of CBD. Atsmon et al. [26] developed gelatin soft capsules containing CBD based on the SNEDDS technology, and the relative bioavailability and Cmax were increased by 0.3- and 1.6-fold, and the Tmax was shorted from 3.5 h to 1.3 h, compared with the reference oromucosal spray. Knaub et al. [21] designed a liquid formulation based on the VESIsorb^®^ SEDDS technology, which could significantly increase Cmax (a 4.4-fold increase as compared to the CBD-MCT) and favorably improve bioavailability (AUC) with rapid absorption. Recently, Kok et al. [22] further developed CBD-loaded SNEDDS formulations. The pharmacokinetic studies in rats indicated that SNEDDS formulations resulted in faster and more significant absorption when compared to the two MCT and sesame oil-based formulations.

Traditionally, SNEDDSs are typically encapsulated in hard or soft gelatin capsules (for example Gengraf^®^, Lipirex^®^, Sandimmune^®^, and Neoral^®^). Although the liquid SNEDDS can improve the oral absorption of hydrophobic drugs, there are still many factors restricting its application in clinical practice, such as precipitation under low temperatures, a low drug loading capacity, drug leakage from capsules, the incompatibility of excipients-capsule and stability problems in long-term storage and transportation [27,28].

Transforming the liquid SNEDDS into the solid state can overcome the deficiencies of SNEDDSs, which has attracted great attention and interest. Solid SNEDDSs (S-SNEDDSs) can be fabricated using solid carrier adsorption, spray drying, hot melt extrusion, and freeze drying techniques [29]. S-SNEDDS formulations combine the advantages of the L-SNEDDSs and solid forms. They can result in freely flowing powders, and further develop into more solid dosage forms like granules, dry suspensions, tablets, and capsules, thereby bringing the benefits of precise dosing, controllable release behavior, easy administration, good patient compliance, good stability, and facilitating storage and transportation [27,30]. However, there are relatively few studies on the solid SNEDDSs of CBD [31].

Consequently, the primary objective of this study is to carry out comprehensive examinations to evaluate the practical feasibility of the L-SNEDDS and the S-SNEDDS in enhancing the oral bioavailability of CBD. Firstly, L-SNEDDS formulations were investigated in detail to improve the solubility and drug release of CBD, and the optimal L-SNEDDS was obtained for subsequent solidification research. Secondly, the solid SNEDDS formulations were prepared using solid carrier adsorption and spray drying techniques, and detailed in vitro characterization techniques were investigated. Additionally, the in vivo evaluation of the optimized L-SNEDDS and S-SNEDDS formulations was investigated in Sprague Dawley rats, and pharmacokinetic parameters were compared with the two oil-based formulations (CBD-MCT and CBD-sesame oil).

## 2. Materials and Methods

### 2.1. Materials

CBD (purity 99%) was gifted from Topharman (Weifang, China). Medium-chain triglyceride (MCT) was donated from IOI Oleo (Hamburg, Germany). Maisine CC, Transcutol HP, Labrasol, Capryol 90, Lauroglycol 90, Labrafil M 1944CS, Labrafil M 2125CS, Maisine CC, Peceol, and Plurol Oleique were generously gifted from Gatteffosse (Lyon, France). Poloxamer 407, PVP K25, PVP K30, and PVP VA64 were kindly gifted from BASF (Ludwigshafen, Germany). Soybean oil, sesame oil, and olive oil were kindly gifted from Société (Saint-Laurent-Blangy, France). SYLOID 244FP, XDP 3050, and XDP 3150 were gifted from Grace (Columbia, MD, USA). Fujicalin SG, Neusilin UFL2, and Neusilin US2, were generously gifted from Fuji (Toyama, Japan). DI-CAFOS A150 was gifted from Budenheim (Budenheim, Germany). Aerosil 200 was purchased from Evonik (Essen, Germany). Calcium silicate was kindly gifted from Kirsch (Salzgitter, Germany). Microcrystalline cellulose (MCC), hydroxy propyl cellulose (HPC), croscarmellose sodium, and magnesium stearate were purchased from Sunhere (Huainan, China). Other reagents and materials were obtained from Macklin (Shanghai, China).

### 2.2. Method for Quantification of CBD

HPLC (Ulti Mate 3000, Thermo Fisher, Waltham, MA, USA) was equipped with a UV-vis detector to quantify CBD. A ZORBAX SB-C18 (4.6 × 150 mm, 3.5 µm, Agilent, Santa Clara, CA, USA) column was employed for the separation of analytes. A mixture of 80% (*v*/*v*) HPLC grade acetonitrile and 20% (*v*/*v*) water was adopted as the mobile phase in the isocratic elution mode, where the flow rate was set at 1.0 mL/min. The column oven temperature was adjusted to 30 °C. The total time for analysis was 7 min per run. A 10 µL sample solution volume was injected with a wavelength of 210 nm.

### 2.3. Development of Liquid SNEDDS (L-SNEDDS)

#### 2.3.1. Screening Solubility of CBD

An excess amount of CBD was added to 2 mL of each excipient. To acquire a supersaturated solution, the mixture was agitated for 24 h in a water bath at 37 °C with a speed of 50 rpm. After centrifuging the sample at 10,000× *g* for 10 min, the supernatant was diluted with acetonitrile to an appropriate concentration, and then filtered through a 0.45 μm PTFE filter membrane. The drug concentration was evaluated using the HPLC method.

#### 2.3.2. Construction of Pseudo-Ternary Phase Diagram

To observe the excipients of SNEDDS, a pseudo-ternary phase diagram was fabricated by employing the aqueous titration approach involving oil, water, and the mixtures of surfactant and co-surfactant. Each represents an apex of the triangle [32,33].

The oil and the mixtures of surfactant and co-surfactant were mixed with various ratios (1:9, 2:8, 3:7, 4:6, 5:5, 6:4, 7:3, 8:2, 9:1) in individual glass vials and stirred at 50 rpm. Subsequently, they were titrated with purified water drop by drop, and a magnetic stirrer was used after each addition under room temperature. Any turbidity or phase alterations were noted, and the mass of water was documented. The pseudo-ternary phase diagrams were created using the Origin Pro 2018 v9.5 software to obtain the nanoemulsion region.

Based on the solubility data for CBD, the oil (MCT), two surfactants (the ratios of Labrasol and Tween 80), and a co-surfactant (Transutol) were screened by pseudo-ternary phase diagrams.

#### 2.3.3. Preparation of CBD-Loaded SNEDDS Formulations

Blank SNEDDS formulations were prepared by accurately weighing the oil, surfactant, and co-surfactant, according to the pre-determined ratio of the prescriptions (the total amount of each prescription was 10 g), and then mixed with a magnetic stirrer at 50 rpm for 30 min in a 25 mL beaker.

The CBD-loaded SNEDDS formulations were prepared by adding 3% CBD to the blank SNEDDS formulations (*w*/*w*, 0.3 g CBD/ 10 g blank SNEDDS), and stirring for 30 min at 50 rpm. Finally, the drug-loaded SNEDDS formulations became clear and transparent.

#### 2.3.4. Preparation of Sesame Oil-Based/MCT-Based CBD Formulations

The CBD-MCT formulation was prepared by adding 0.3 g of CBD into a 25 mL beaker with 10 g MCT oils, then stirred for 30 min at 50 rpm.

To remain in line with the CBD concentration of the SNEDDS, we slightly adjusted the sesame oil formulation by referring to the patent prescription [34]. The CBD-sesame oil formulation was prepared by adding 3.0 g of CBD, 7.9 g of ethanol, 50 mg of sucralose, 20 mg of strawberry flavoring, and 92.03 g of sesame oil into a 200 mL beaker, and then stirred for 30 min at 50 rpm.

#### 2.3.5. Characterization of CBD-Loaded SNEDDS Formulations

##### Droplet Size and PDI Value

To measure the droplet size and PDI of the SNEDDS formulations, the samples were diluted 1000-fold in purified water and analyzed using a size and Zeta analyzer (BeNano 90, Bettersize, Dandong, China). Triple measurements were carried out to ensure the accuracy and reproducibility.

##### In Vitro Drug Release Profile

The dissolution profiles of CBD-MCT, CBD-sesame oil, and CBD-loaded L-SNEDDS formulations were investigated through a USP 29 apparatus II with a dissolution tester (AT. 7X, Sotax, Switzerland). During the entire testing process, the dissolution medium was sustained at 37 ± 0.5 °C, and the velocity of the paddle was set at 100 rpm. The formulations containing 30 mg of CBD equivalent were subjected to 900 mL of simulated gastric fluids (pH 1.0) or simulated intestinal fluids (the phosphate buffer of 6.8). At pre-defined time points (15, 30, 45, and 60 min), an aliquot (1 mL) of the sample was removed from the dissolution medium and filtered via a 0.45 μm PES filter membrane. Each aliquot was then analyzed by HPLC as previously described. Measurements were performed in triplicates.

#### 2.3.6. Storage Stability of CBD-Loaded L-SNEDDS

The selected CBD-loaded L-SNEDDS-F01 formulations were sealed in an ampoule. For the storage stability test, the samples were placed under diverse storage conditions (such as −20 °C, 4 °C, 25 °C, and 37 °C) for incubation. At the pre-defined time intervals, the samples were gathered and then diluted 1000 times with purified water, and then the droplet size and PDI value of the diluted samples were measured.

#### 2.3.7. Investigation on the Ability of SNEDDS Loading CBD

The loading ability of the SNEDDS for CBD is significant, which offers a foundation for prescription screening and optimization, and is conducive to develop a drug-loading system with better performance. According to the formulation of SNEDDS-F01, drug-loaded SNEDDS were prepared by adding 30 mg, 60 mg, 90 mg, and 120 mg CBD per gram of SNEDDS, respectively. The solubility was visually observed, and the SNEDDS formulations were characterized following the method in Section 2.3.5.

#### 2.3.8. Transmission Electron Microscopy (TEM)

To observe the surface morphology, the L-SNEDDS-F07 was diluted 1:1000 with purified water, and the sample was dropped onto the TEM grid (carbon coated, 200 mesh copper grid). Afterward, a 1% (*w*/*v*) phosphotungstic acid solution was employed to color the copper grids for 4 min. Then, the dyed grids were dried and inspected by the TEM (HT7700, 100 kV, Hitachi, Tokyo, Japan). The images were recorded on film at a magnification of 10,000 times.

### 2.4. Development of CBD-Loaded Solid SNEDDS by Solid Carrier Adsorption (SCA) Method

#### 2.4.1. Inspection of Oil Adsorption Capacity of Solid Carriers

Solid SNEDDS (S-SNEDDS) formulations were achieved by adsorbing the CBD-loaded liquid SNEDDS formulation (L-SNEDDS-F07) into solid carriers. Solid carriers such as magnesium aluminum metasilicate, silicon dioxide, and anhydrous dibasic calcium phosphate were studied. The oil adsorption capacity was determined for each solid carrier. One gram of each carrier was placed independently in a mortar. The formulation L-SNEDDS-F07 was added dropwise and mixed well after each addition until a non-sticky powder flowing freely was obtained, and the weight of the L-SNEDDS-F07 formulation used was recorded [35].

#### 2.4.2. Preparation of CBD-Loaded S-SNEDDS

The CBD-loaded S-SNEDDS formulations were prepared by using three carrier materials of Neusilin UFL2, SYLOID XDP 3150 (the ratio of carriers and L-SNEDDS was 1:1), and Fujicalin SG (the ratio of carrier to L-SNEDDS was 2:1), respectively. In this study, 20 g of each carrier was placed separately in a mortar, and the L-SNEDDS formulations were added dropwise. The mixed powder was added by filler (MCC), binder (HPC), disintegrant (croscarmellose sodium), and lubricant (magnesium stearate).

#### 2.4.3. Scanning Flow Properties and Compressibility of S-SNEDDS

The flow properties of the S-SNEDDS powders were determined using Carr’s index and angle of repose (θ).

The weighed quantity of powder (4 ± 0.2 g) was precisely added to a graduated 25 mL cylinder to determine the bulk and tapped densities. Subsequently, the cylinder was tapped 500 times until there was no alteration in volume. Carr’s index was calculated in line with Equation (1) [36]. Every measurement was conducted in triplicate.

Carr’s index = 100 [(tapped density − bulk density)/tapped density](1)

Serving as a critical index for assessing the flow characteristics of the S-SNEDDS powders, the angle of repose indicates the maximal angle of the horizontal plane of a conical heap of particles. To measure the angle of repose, the S-SNEDDS powders were steadily poured via a funnel into a beaker approximately 3 cm beneath the funnel’s spout. Once the particles were stabilized, the height (h) and the base diameter (d) of the formed cone were noted. Thereafter, the angle of repose (θ) was determined through the employment of these measurements in accordance with the following Equation (2) [37,38].

θ = tan^−1^ (2h/d) (2)

The S-SNEDDS powders were compacted into tablets using a rhombic tableting mold of 14.4 × 10.4 mm (average tablet weight was 1 g, with a weight variation of ±5%). The appearance characteristic of tablets was observed to investigate the compressibility of the powders.

#### 2.4.4. In Vitro Drug Release Profile and Nanoemulsion Study

The dissolution parameters can refer to the parameters under Section 2.3.5 “In Vitro Drug Release Profile”. CBD-loaded SNEDDS formulations with 25 mg of CBD equivalent were subjected to 900 mL of simulated gastric fluids (distilled water at pH 1.0). Measurements were performed in triplicates.

To investigate the nanoemulsions, 10 mL of the liquid sample was taken at the dissolution endpoint of 1 h, and filtered through a 0.45 μm PES filter membrane, and then the droplet size and PDI value were studied.

### 2.5. Development of CBD-Loaded S-SNEDDS by Spray Drying (SD) Method

#### 2.5.1. Investigation of Precipitation Inhibitors (PI)

PVP K25, PVP K30, PVP VA64, and Poloxamer 407 were evaluated as PI. Experiments were carried out by adding 3% (*w*/*w*) of the mentioned polymers to the prepared L-SNEDDS-F07 formulation. Afterward, the mixtures were dissolved utilizing magnetic stirring, the dissolution state of the sample was observed, and the PI was chosen to be completely dissolved in the CBD-loaded L-SNEDDS.

#### 2.5.2. Preparation of CBD-Loaded S-SNEDDS by SD Method

The S-SNEDDS formulations were prepared through a spray dryer (ADL311S, YAMATO, Tokyo, Japan). Silicon dioxide (Aerosil 200) and calcium silicate were selected as the carriers for the S-SNEDDS (the weight ratio of the carrier to L-SNEDDS was 1:1). PVP VA64 (3.0% ratio of the L-SNEDDS) was selected as PI. Meanwhile, the influence of adding or not adding PVP VA64 in the spray drying of calcium silicate was investigated.

Solid carrier and PVP VA64 were suspended in the 10% ethanol solution. After that, the CBD-loaded L-SNEDDS-F07 was added to the solution under vigorous stirring conditions. The blend was continuously stirred to keep a steady suspension status during spray drying. The spray drying operation was performed under the conditions where the inlet temperature was 100 °C, the outlet temperature was 50 °C, the drying air blower rate was 0.40 m^3^/min, and the spray pressure was 100 kPa [39].

#### 2.5.3. Scanning Flow Properties of S-SNEDDS

The research of flowability could refer to the method in Section 2.4.3.

#### 2.5.4. In Vitro Drug Release Profile and Nanoemulsion Study

The dissolution and nanoemulsion parameters could refer to the method under Section 2.4.4.

#### 2.5.5. Scanning Particle Size of CBD-Loaded S-SNEDDS-SD Powders

The particle size distributions of the carrier of the calcium silicate and the S-SNEDDS-F13 (SD powders) were determined by laser diffraction of a Mastersizer 3000 (Malvern Instruments, Malvern, Worcestershire, UK). The method was as follows: dry method, hopper gap 2.00 mm, air pressure 2.4 bar, injection rate 39%, and high energy venturi tube. Triplicate measurements were conducted to ensure the accuracy and reproducibility, and each value is represented as the mean ± S.D.

### 2.6. Preparation and Characterization of Laboratory-Scale S-SNEDDS Powders

#### 2.6.1. Preparation of Laboratory-Scale S-SNEDDS Powders

Through the above investigations, the types of solid carriers were determined. The CBD-loaded S-SNEDDS via solid carrier adsorption method was prepared by utilizing Fujicalin SG (the ratio of carrier to L-SNEDDS was 2:1). The CBD-loaded S-SNEDDS via spray drying method was prepared by calcium silicate and PVP VA64. The prescription and the spray drying process parameters were detailed under Section 2.5.2.

#### 2.6.2. Scanning Flow Properties of S-SNEDDS Powders

The research of flowability could refer to the method under Section 2.4.3.

#### 2.6.3. In Vitro Drug Release Profile

The dissolution parameters could refer to the parameters under Section 2.4.4. CBD-loaded SNEDDS formulations with 5 mg of the CBD equivalent were subjected to 900 mL of simulated gastric fluids (distilled water at pH 1.0) and simulated intestinal fluids (the phosphate buffer of pH 6.8). Measurements were performed in triplicates.

#### 2.6.4. X-Ray Diffraction (XRD)

The XRD of pure CBD, carriers, and drug-loaded S-SNEDDS formulations were studied with the utilization of Bruker D8 Advance Focus P-XRD (Billerica, MA, USA) with Cu-Kα radiation (40 mA, 40 kV) at 2-θ angles of 10–40°, with a slow angle scan of 0.01°/min at a sampling interval of 0.02°/s [40].

#### 2.6.5. Differential Scanning Calorimetry (DSC)

CBD, solid carriers, blank S-SNEDDS and CBD-loaded S-SNEDDS powders were analyzed using a differential scanning calorimeter (DSC-3, Mettler Toledo, Greifensee, Switzerland). Samples within the range of 2 to 5 mg were maintained in aluminum pans. The heat was exerted upon the pans with a temperature range from 40 to 250 °C, with a heating rate of 10 °C per minute, and beneath nitrogen gas (the flow rate of 50 mL/min).

#### 2.6.6. Fourier Transform Infrared Spectroscopy (FTIR)

The FTIR scans for pure CBD, blank S-SNEDDS, and CBD-loaded S-SNEDDS were performed using the FTIR spectrophotometer instrument (Nicolet™ iS™5, Thermo Fisher, Waltham, MA, USA). Powder samples were combined with potassium bromide in a ratio of 1:100, and then compressed via a hydraulic press to form disks, which thereafter underwent scanning within the range from 4000 to 500 cm^−1^.

#### 2.6.7. Scanning Electron Microscopy (SEM)

The surface morphologies of CBD, Fujicalin SG, calcium silicate, and CBD-loaded S-SNEDDS formulations were examined using an SEM (Gemini 300, Zeiss, Oberkochen, Germany). Before the observation the samples were held on an aluminum dock using a double-sided adhesive tape, which was further covered with gold (~20 nm) to render it electrically conductive in a vacuum. The scanning action of the SEM was carried out at an acceleration voltage of 2 kV.

### 2.7. Preparation and Characterization of S-SNEDDS Unit Dose Formulations

#### 2.7.1. Preparation of S-SNEDDS Unit Dose Formulations

Based on the “Preparation of laboratory-scale S-SNEDDS powders” study in Section 2.6.1, the S-SNEDDS powders were added with MCC, croscarmellose sodium, and magnesium stearate for homogeneous mixing. Unit dose formulations such as tablets and capsules were further prepared. The tablet press mold was a 7 mm circular shallow concave punch, and the hard gelatin capsule shell was a size zero hard capsule shell.

#### 2.7.2. In Vitro Drug Release Profile and Nanoemulsion Study

The method could refer to the dissolution parameters under Section 2.4.4. CBD-loaded SNEDDS formulations with 5 mg of a CBD equivalent were subjected to 900 mL of pH 1.0 and pH 6.8 medium. Measurements were performed in triplicates.

#### 2.7.3. Stability Study

In order to assess the performance upon storage in accelerated conditions, the capsules of S-SNEDDS-SCA and S-SNEDDS-SD were enrolled in stability investigations. The formulations were placed in a stability cabinet (SHH-400SD, YSEI, Chongqing, China) with accelerated conditions (40 ± 2 °C, RH 75 ± 5%). At pre-arranged intervals (1 month and 2 months), samples were taken out and permitted to reach ambient temperature. The drug release in pH 1.0, XRD, and DSC studies were carried out to assess the change in drug release and drug crystallinity during storage [41,42].

### 2.8. In Vivo Pharmacokinetic Study

#### 2.8.1. Animals

Healthy 7–8-week-old male Sprague Dawley rats weighing 200–280 g were housed in appropriate cages. Animals were maintained under standard laboratory conditions with a regular 12 h day-night cycle in well-ventilated rooms with an average temperature of 23 ± 2 °C and a relative humidity of 40–50%. The animal study protocol was approved by the Experimental Animal Ethics Committee of Shanghai Shengchang Biotechnology Co., Ltd. (Shanghai, China, protocol code: 2024-01-CZ-LY-055 and date of approval: 1 January 2024).

#### 2.8.2. In Vivo Pharmacokinetic Study

Animals were divided into five groups, each consisting of three rats (except for four rats in the CBD-sesame oil formulation). The five formulations were CBD-MCT, CBD-sesame oil, L-SNEDDS, S-SNEDDS-SCA, and S-SNEDDS-SD. CBD-MCT and CBD-sesame oil were prepared using the method under Section 2.3.4. L-SNEDDS (L-SNEDDS-F01) was prepared under Section 2.3.3. S-SNEDDS-SCA (S-SNEDDS-F14) and S-SNEDDS-SD (S-SNEDDS-F15) were prepared under Section 2.6.1. The five formulations were orally administered to the rats at a dose of 15 mg/kg using a gavage needle. CBD-MCT and CBD-sesame oil were dispersed in MCT and sesame oil, respectively. L-SNEDDS, S-SNEDDS-SCA, and S-SNEDDS-SD were dispersed in purified water. Compared with humans, the amount of liquid in the stomach of rats is smaller, and the gastric juice of rats is insufficient to cause the self-emulsification of SNEDDS formulations [23,43]. Hence, both liquid and solid SNEDDS formulations were dispersed in water before administration to undergo spontaneous emulsification. However, there were also certain limitations in administering the solid SNEDDS dispersed in water, as it could not reflect the disintegration process of solid preparations in the stomach.

The rats underwent fasting for 12 h before the administration, and food was given after 6 h of drug administration. Blood samples of 0.2 mL were collected from the jugular vein at 0.08, 0.25, 0.5, 1, 2, 4, 6, 8, and 24 h after dosing (except for the CBD-sesame oil formulation of adding blood samples at 10 h, 12 h). The blood samples were placed into heparin anticoagulant tubes, centrifuged at 11,000 rpm for 5 min under the temperature of 4 °C, and the resulting plasma samples were frozen at −70.0 °C before being analyzed using the LC-MS/MS method.

#### 2.8.3. Bioanalytical Method

The plasma samples were analyzed using the LC-MS/MS method. The internal standard solution was verapamil (200 ng/mL, diluted in 50% ethanol solution). 20 μL of plasma was vortex-mixed for 10 min with 200 μL of acetonitrile and 20 μL of verapamil solution. Then, it was separated by centrifugation (3900 rpm, 10 min, 4 °C). 150 μL of the supernatant was transferred to the clean 96-well plate, and 150 μL of purified water was added. After thorough mixing, the sample was ready for injection and analysis. The content was determined using a high-performance liquid chromatography (HPLC) system (LC-30AD, Shimadzu, Kyoto, Japan) with a mass-spectrometer (TRIPLE QUAD 5500, Applied Biosystems, Concord, ON, Canada). The HPLC—MS conditions were as follows: ZORBAX 300 SB-C8 (4.6 × 50 mm, 5 µm, Shimadzu, Kyoto, Japan), an isocratic mobile phase composed of solutions A (methanol) and B (1 mM ammonium acetate buffer containing 0.2% formic acid) (6/4, *v*/*v*), and a flow rate of 0.6 mL/min at 40 °C. CBD and verapamil were detected in positive ion mode. The CBD detection masses (*m*/*z*) were from 315.2 to 193.3, and the verapamil detection masses (*m*/*z*) were from 455.2 to 164.9. The ion source temperature was 500 °C, the spray voltage was 5500 V, the nebulizer gas was 65 psi, the auxiliary gas was 60 psi, and the curtain gas was 30 psi. The linearity for CBD was between 2.00 and 5000 ng/mL, with a correlation coefficient of 0.9950.

#### 2.8.4. Data Analysis

Using the WinNonlin^®^ software (version 8.1, Pharsight, Mountain View, CA, USA), non-compartmental analysis was used to calculate various pharmacokinetic parameters. These included the area under the plasma concentration versus time curve (AUC_0–4 h_, AUC_0–24 h_, and AUC_0–∞_) and the half-life (T_1/2_). The maximum plasma concentration (Cmax) and the time required to reach Cmax (Tmax) were directly obtained from the plasma data.

The experimental results were expressed as the means ± SEM. Statistical comparison of data was performed by the two-tailed unpaired Student’s *t*-test using GraphPad Prism 9.5.1 software. A *p*-value of less than 0.05 is considered to be significant.

## 3. Results and Discussion

### 3.1. Development of L-SNEDDS

#### 3.1.1. Screening Solubility of CBD

Conducting the screening for the drug solubility in oils, surfactants, and co-surfactants constitutes a fundamental prerequisite for the optimization of the components within the SNEDDS formulations [28].

**Figure 1:**
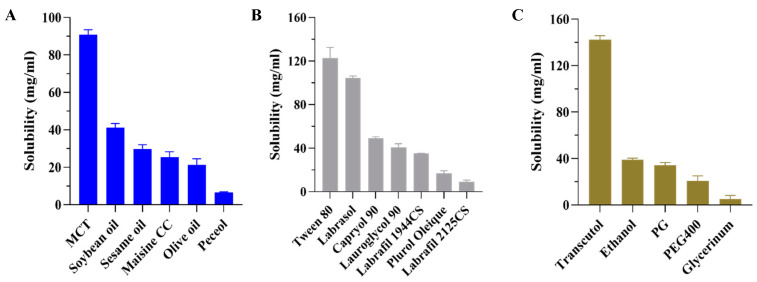
Solubility data of CBD in (**A**) oils, (**B**) surfactants, and (**C**) co-surfactants. Each value is represented as the mean ± S.D. (*n* = 3).

Among the six oils (Figure 1A), it was found that CBD was most soluble in MCT (90.8 ± 2.6 mg/mL), followed by soybean oil, sesame oil, Maisine CC, olive oil, and Peceol. MCT was found to be more advantageous in dissolving CBD than the plant oils (such as soybean oil, sesame oil, and olive oil). Since the oil component serves as the primary reservoir for lipophilic drugs in SNEDDSs, MCT, which exhibited the highest solubility for CBD, was selected as the oil phase.

Among the seven surfactants (Figure 1B), CBD was most soluble in Tween 80 (122.7 ± 9.8 mg/mL), followed by Labrasol (104.4 ± 1.9 mg/mL), Capryol 90, Lauroglycol 90, Labrafil 1944CS, Plurol Oleique CC497, and Labrafil M 2125CS. CBD had relatively good solubility in both Tween 80 and Labrasol. Tween 80 and Labrasol, non-ionic surfactants with high HLB (15 and 12, respectively), can spontaneously generate transparent emulsions. Labrasol and Tween 80, which have a low toxicity and cause less harm to human health and the environment, are the preferred surfactants for oral administration.

Among the five co-surfactants (Figure 1C), CBD exhibited the highest solubility in Transcutol (142.3 ± 3.4 mg/mL), followed by Ethanol, Propylene glycol (PG), PEG400, and Glycerinum. Transcutol dissolved a much more significant amount of CBD than the others. Transcutol is a capable solubilizer with a low HLB value of 4.2, which helps to stabilize the interfacial film, reduces surface tension, and enhances emulsification effect in conjunction with the surfactant [35,36].

Consequently, owing to the results of saturation solubility of CBD, MCT, Labrasol, Tween 80, and Transcutol were selected as the oil, mixed surfactants, and co-surfactants for the SNEDDS formulations, respectively.

#### 3.1.2. Construction of Pseudo-Ternary Phase Diagram

The pseudo-ternary phase diagrams were fabricated to label the self-nanoemulsifying regions of the different Smix of the mixed surfactants (Smix, the ratios of Labrasol and Tween 80) formulations. Emulsification efficiency is an additional critical parameter in the screening of SNEDDS prescriptions. A more extensive nanoemulsion region implies a greater nanoemulsification efficiency [36].

**Figure 2:**
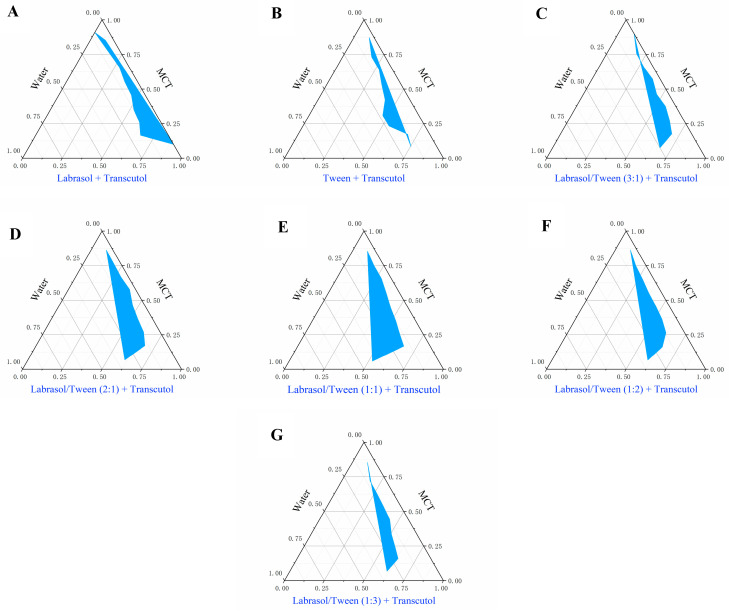
Pseudo-ternary phase diagrams of different ratios of mixed surfactants (Labrasol and Tween 80): (**A**) only Labrasol, (**B**) only Tween 80, (**C**) 3: 1, (**D**) 2: 1, (**E**) 1: 1, (**F**) 1: 2, and (**G**) 1: 3. The region with color signified the nanoemulsion area (*n* = 3).

The nanoemulsion regions of pseudo-ternary phase diagrams are shown in Table 1 and Figure 2A–G. We screened Labrasol and Tween 80 as the mixed surfactants with similar relatively high solubility to draw the pseudo-ternary phase diagram. Compared to a single surfactant, the employment of mixed surfactants could increase the region of emulsion formation. When utilized as mixed surfactants in a ratio of 1:1, the emulsion region turned out to be relatively large. Hence, Labrasol and Tween 80 were selected as mixed surfactants with a ratio of 1:1.

#### 3.1.3. Preparation of CBD-Loaded SNEDDS Formulations

Based on the result of saturated solubility and the pseudo-ternary phase diagrams, the four SNEDDS formulations loaded with CBD were fabricated. CBD is typically dissolved in oil solutions due to its high lipophilic nature. Herein, two oil-based formulations of sesame oil (in accordance with Epidiolex^®^, an approved CBD drug by the US FDA) and MCT were prepared as the control groups for the SNEDDS formulations. The prescription design is presented in Table 2.

#### 3.1.4. Characterization of CBD-Loaded SNEDDS

**Figure 3:**
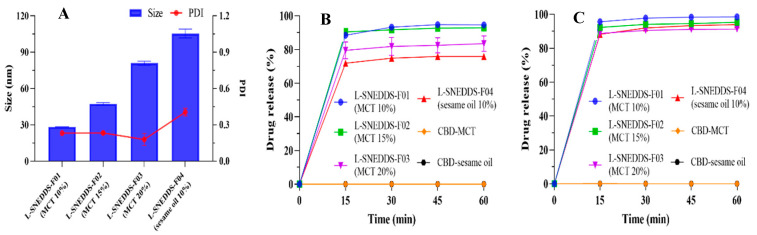
Characterization of the CBD-loaded SNEDDS formulations. (**A**) The droplet size and PDI value, the dissolution profile in (**B**) pH 1.0 and (**C**) pH 6.8 conditions of different oils formulations (mean ± S.D., *n* = 3).

The droplet size and PDI value of the CBD-loaded SNEDDS formulations are depicted in Figure 3A. As the proportion of MCT increased from 10% to 20%, the droplet size of SNEDDS increased accordingly (from 28.2 ± 0.3 nm to 80.9 ± 1.7 nm). By comparing the two SNEDDS formulations utilized by MCT and sesame oil, the droplet size of the sesame oil SNEDDS was larger, and it had a greater PDI value (0.405 ± 0.032), indicating an unstable state. The increase in the oil phase ratio leads to a decrease in the overall density of the emulsion, thereby facilitating the coalescence of lipid droplets to form larger particle diameters. Sesame oil is a complex natural vegetable oil. Compared to MCT, due to its relatively larger molecular weight, complex composition, higher viscosity, and lower solubility, the nanoemulsion formed by sesame oil had a relatively large droplet size and poor stability. The SNEDDS formulation with a 10% MCT usage exhibited a smaller droplet size and better stability.

Figure 3C revealed no remarkable change in drug release among different SNEDDS formulations under pH 6.8 conditions. As shown in Figure 3B, compared with the SNEDDS formulation with 10% MCT, when the proportion of MCT increased to 20%, the dissolution of SNEDDS in the pH 1.0 medium was decreased by approximately 10% (from 94.59 ± 0.66% to 83.48 ± 4.59% in 1 h). When the oil was switched from MCT to sesame oil, the dissolution in the hydrochloric acid medium was lower (from 94.59 ± 0.66% to 75.79 ± 1.41% in 1 h). It was inferred that this appearance was related to the relatively large granularity of the SNEDDS formed by sesame oil and the low solubility of CBD in sesame oil, which led to a comparatively low self-emulsifying ability in the stomach. In contrast to SNEDDS, the CBD oil-based solutions of MCT and sesame oil had a dissolution of less than 1% in the medium under pH 1.0 and 6.8 conditions. The oil-based formulations (sesame oil or medium-chain triglyceride) were immiscible with the water. The oily droplets were also observed during the dissolution experiment, which led to the incapability of the hydrophobic CBD to dissolve. SNEDDS formulations contain surfactants and co-surfactants, which reduce the oil-water interfacial tension and enable the emulsion to form smaller droplets. When exposed to an aqueous solution, SNEDDS can spontaneously emulsify to generate nano-droplets with particle sizes of less than 200 nm. The spontaneous emulsification process eventually results in a reduction in particle size and maximizes surface area. Thus, nanoemulsion can significantly enhance the drug release behavior. Based on the above research, the L-SNEDDS-F01 (10% MCT) was given for subsequent research.

#### 3.1.5. Storage Stability of CBD-Loaded L-SNEDDS Formulation

**Figure 4:**
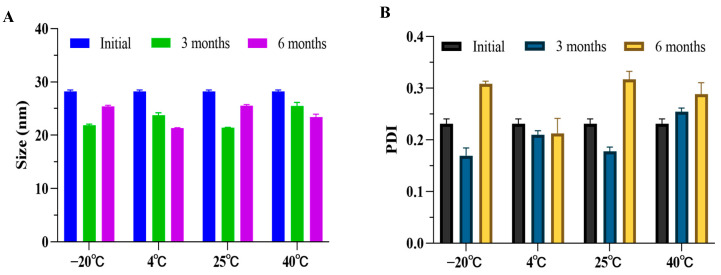
Storage stability of the CBD-loaded SNEDDS formulations over six months under different storage conditions. (**A**) The droplet size and (**B**) PDI value (mean ± S.D., *n* = 3).

The storage stability is shown in Figure 4. The CBD-loaded L-SNEDDS-F01 formulation was stored over six months under the storage conditions of −20 °C, 4 °C, 25 °C, and 40 °C. As the storage time increased, there was no significant change in the droplet size of the L-SNEDDS samples under different storage conditions. Compared with the initial SNEDDS, the PDI value of the samples stored for 6 months tended to increase slowly. Under the storage condition of 4 °C, the droplet size and the PDI value were the smallest, and the samples were the most stable.

#### 3.1.6. Investigation on the Ability of SNEDDS Loading CBD

**Figure 5:**
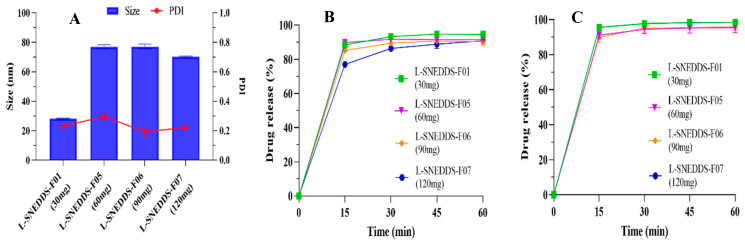
Characterization of the SNEDDSs loading different amounts of CBD. (**A**) The droplet size and PDI value, the dissolution profile in (**B**) pH 1.0 and (**C**) pH 6.8 (mean ± S.D., *n* = 3).

The currently developed SNEDDS formulation exhibits an excellent capacity for drug loading, and up to 120 mg of CBD could be fully dissolved in each gram of SNEDDS. As depicted in Figure 5, it was noted that the droplet size increased (from 28.2 ± 0.3 nm to 76.9 ± 1.5 nm) as the amount of the loaded CBD increased from 30 mg to 60 mg. Interestingly, the SNEDDS formulations loaded with 60 mg to 120 mg CBD had similar granularity (70.3 ± 0.4 nm to 76.9 ± 1.5 nm) and low PDI values (0.194 ± 0.036 to 0.295 ± 0.014). The formulations with varying drug loading amounts (ranging from 30 mg to 120 mg) could completely dissolve in the pH 1.0 and 6.8 medium. This suggested that the SNEDDS formulations possessed relatively good self-emulsifying ability and dissolution capacity, and could load a relatively large amount of CBD (≥120 mg).

#### 3.1.7. TEM

**Figure 6:**
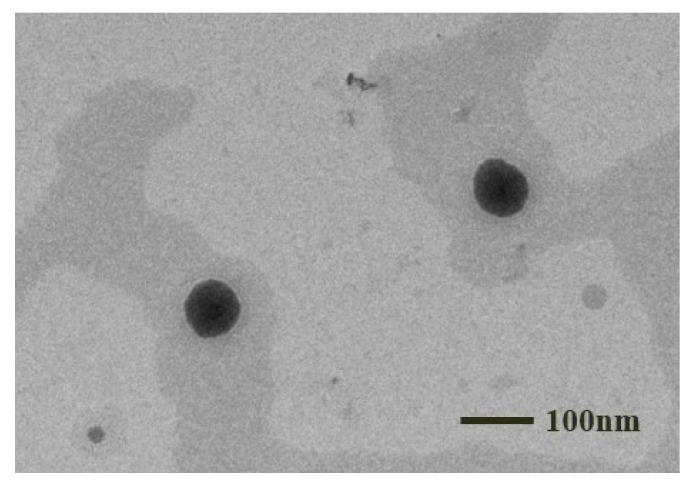
TEM image of L-SNEDDS-F07 formulation.

The TEM image is presented in Figure 6. The nanoemulsion globules of the selected L-SNEDDS-F07 for subsequent studies were spherical. They showed no sign of droplet agglomeration, consistent with the findings obtained from dynamic light scattering (the droplet size: 70.3 ± 0.4 nm; PDI value: 0.220 ± 0.000), indicating a homogeneous dispersion formulation.

### 3.2. Development of CBD-Loaded S-SNEDDS by Solid Carrier Adsorption (SCA) Method

#### 3.2.1. Inspection of Oil Adsorption Capacity of Solid Carriers

In recent years, adsorption onto solid carriers has emerged as the most intensively investigated approach for obtaining solid SNEDDS formulations. Solid carriers such as silicon dioxide, magnesium aluminum metasilicate, and anhydrous dibasic calcium phosphate are considered safe and effectively utilized to produce solid SNEDDS [44].

The oil adsorption capacity of different carriers is shown in Table 3. The comparison regarding the oil adsorption capacity of carriers revealed that: magnesium aluminum metasilicate > silicon dioxide > anhydrous dibasic calcium phosphate. Among the three types of silicon dioxide, SYLOID XDP 3150 possessed a relatively better oil adsorption capacity. The oil adsorption capacity of Neusilin UFL2 was slightly better than that of Neusilin US2. Among the two types of anhydrous dibasic calcium phosphate, Fujicalin SG exhibited a stronger oil adsorption capacity.

In conclusion, SYLOID XDP 3150, Neusilin UFL2, and Fujicalin SG were preferentially selected for the subsequent investigation, respectively.

#### 3.2.2. Preparation of CBD-Loaded S-SNEDDS

In this study, S-SNEDDS formulations were prepared by employing three carriers of Neusilin UFL2, SYLOID XDP 3150, and Fujicalin SG, following the ratios presented in Table 4. The flow characteristics of the mixed powders were determined through Carr’s index and the angle of repose. Subsequently, the mixed powders were combined with filler, binder, disintegrant, and lubricant substances for tablets, and the compressibility of these materials was examined.

#### 3.2.3. Scanning Flow Properties and Compressibility of S-SNEDDS

The investigation of the flow properties of the powders plays a significant role in the development of solid formulations. Carr’s index and angle of repose are two parameters utilized to evaluate the flowability of powders. A Carr’s index value beneath 25% implies favorable flow properties, while a Carr’s index exceeding 25% indicates poor flowability [45,46]. In contrast, when the angle of repose of powder is lower than 40°, it can meet the requirements for flow properties in the processing and production process. Conversely, the flow property is relatively poor if it is greater than 40° [47].

The results of flow properties and compressibility are shown in Table 4. All the formulations exhibited promising flow properties with Carr’s index values between 11.18% and 15.21%, as well as the angle of repose between 25.78° and 29.74°.

The compressibility of the S-SNEDDS-F8 formulation was relatively poor, and a chipping phenomenon was observed during the tableting process. It was speculated that the occurrence of chipping was related to the inherent nature of the solid carrier itself. SYLOID XDP 3150 is an outstanding glidant featuring a smooth surface. When a large amount of silicon dioxide was employed in the formulation, the adhesion between it and other materials was weak, leading to inferior compressibility. The compressibility of S-SNEDDS-F9 and F10 formulations was acceptable. S-SNEDDS-F10 formulation could add fillers such as MCC and lactose to enhance the flow properties and compressibility further.

#### 3.2.4. In Vitro Drug Release and Nanoemulsion Study

**Figure 7:**
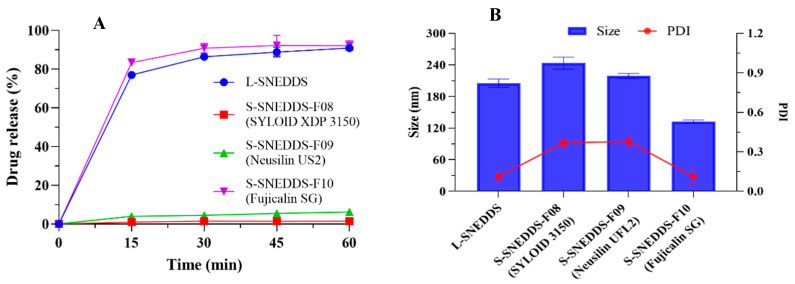
Characterization of S-SNEDDS formulations with different solid carriers in pH 1.0 conditions. (**A**) In vitro drug release, (**B**) droplet size and PDI value. Each value is represented as mean ± S.D. (*n* = 3).

The drug release results in Figure 7 illustrate that different adsorption carriers greatly influenced the in vitro dissolution of SNEDDS.

Both L-SNEDDS and S-SNEDDS-F10 (Fujicalin SG) formulations exhibited comparably rapid CBD release in pH1.0, with approximately 90.88 ± 1.46% and 92.13 ± 2.53% in 1 h, respectively. Meanwhile, the CBD release from S-SNEDDS-F08 (SYLOID XDP 3150) and F09 (Neusilin UFL2) was determined to be 1.48 ± 0.06% and 6.24 ± 1.71% in 1 h. SYLOID XDP 3150 exhibits a layered porous structure with an irregular shape. It has very high inner surfaces (>92%), indicating that the carrier had a lot of porosity inside. Due to the abundant voids and complex channels in the carrier, the drug required to traverse the tortuous pathway before being released, and consequently resulted in a relatively low drug release in S-SNEDDS-F08 (SYLOID XDP 3150) [48]. The average particle size of Neusilin UFL2 is 2–8 μm, which is published by the company of Fuji Chemical. The carrier of Neusilin UFL2 has a relatively small particle size and a large quantity of long and narrow specific pores [35]. CBD was prone to be trapped in these pores, and it was speculated that this was the reason for the low dissolution of CBD in S-SNEDDS-F09 (Neusilin UFL2).

Additionally, the droplet size after the disintegration of the solidified preparation ranged from 100 to 300 nm, and the PDI value was less than 0.4. The droplet size and PDI value of the Fujicalin SG formulation were both relatively low. Combined with the dissolution test, the droplet size, and PDI detection results, Fujicalin SG was used as the adsorption carrier (2:1 *w*/*w* ratio of carrier to L-SNEDDS).

### 3.3. Development of CBD-Loaded S-SNEDDS by Spray Drying (SD) Method

#### 3.3.1. Investigation of Precipitation Inhibitor (PI)

The spray drying process is also a commonly employed method in the solidification research of SNEDDS, which can realize continuous mechanized or automated production, featuring fast drying speed and high production efficiency, and being a process suitable for large-scale commercial production. In the spray drying process, a certain quantity of PI can be added to inhibit drug precipitation and improve the stability and dissolution rate of all SNEDDS formulations.

As shown in Table 5, except for PVP VA64, none of the polymers could be successfully dissolved. Copovidone (PVP VA64) is widely used as an excellent dry binder and carrier material in the spray drying process. The pyrrolidone structure in copovidone has good solubility in water and can provide a solubilization effect for poorly soluble drugs, improving the solubility and release characteristics of the drugs. Therefore, PVP VA64 was selected as PI in this study.

#### 3.3.2. Preparation of CBD-Loaded S-SNEDDS by SD Method

Silicon dioxide and calcium silicate are rather prevalently utilized as spray drying carriers. Hereby, different carriers were employed to solidify L-SNEDDS by the SD method at the ratio of 1:1 (the carrier to L-SNEDDS, *w*/*w*). S-SNEDDS-F11 and F12 formulations were prepared using silicon dioxide (Aerosil 200, Evonik, Essen, Germany) and calcium silicate (Kirsch Pharma, Salzgitter, Germany), respectively. S-SNEDDS-F13 was prepared by adding PVP VA64 (accounting for 3% of L-SNEDDS) based on the F12 formulation to examine the impact of PI. Loose powder could be obtained after the spray drying process of the three prescriptions. The formulation design is detailed in Table 6.

#### 3.3.3. Scanning Flow Properties of S-SNEDDS

The results of the flow properties are shown in Table 6. The S-SNEDDS-F11, F12, and F13 had good flow properties, with Carr’s index values ranging from 20.85% to 21.89%, and the angle of repose between 31.69° and 33.13°.

**Table 6:** Study on the prescriptions and flow properties of the S-SNEDDS with spray drying carriers.

Samples	S-SNEDDS-F11	S-SNEDDS-F12	S-SNEDDS-F13
carrier species	Aerosil 200	calcium silicate	calcium silicate
carrier suppliers	Evonik	Kirsch Pharma	Kirsch Pharma
ingredients	Composition (%)		
solid carrier	50%	50%	50%
L-SNEDDS	50%	50%	50%
PVP VA64	/	/	1.5%
Flow properties of powders			
Carr’s index	21.14 ± 2.35%	21.89 ± 0.68%	20.85 ± 1.94%
the angle of repose	33.13 ± 1.65°	32.86 ± 1.27°	31.69 ± 3.26°

#### 3.3.4. In Vitro Drug Release Profile and Nanoemulsion Study

**Figure 8:**
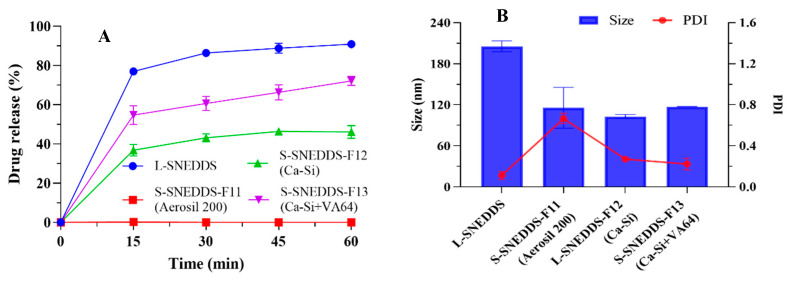
Characterization of the S-SNEDDS formulations using the spray drying method in pH 1.0 conditions. (**A**) In vitro drug release, (**B**) the droplet size and PDI value. Each value is represented as the mean ± S.D. (*n* = 3).

The drug release results are presented in Figure 8. The spray drying carrier had a significant impact on the drug release profile of the S-SNEDDS. The dissolution of S-SNEDDS-F11 with the employment of Aerosil 200 was extremely low (<1%). It was found that the CBD release from S-SNEDDS-F12 (calcium silicate) and F13 (calcium silicate + PVP VA64) was 46.12 ± 3.27% and 72.13 ± 2.12% in 1 h, respectively.

Compared with Aerosil 200, the dissolution result of calcium silicate was better, and the addition of PVP VA64 could enhance the dissolution performance. However, the dissolution of the sample after spray drying curing was lower than that of L-SNEDDS (90.88 ± 1.46% in 1 h). After the cured preparation disintegrated, the droplet size was less than 300 nm. Among these, the PDI value of the Aerosil 200 formulation was relatively large (>0.6), indicating an unstable tendency, while the PDI values of other samples were less than 0.3. Aerosil 200 possesses disordered loose pores and irregular crystal structures. Meanwhile, calcium silicate features a petal-like crystal structure with deep and large porosities on the crystal structure, extending to the structure’s interior [49]. It was speculated that the deep pore channels and specific lattice arrangement of calcium silicate provided more attachment and penetration sites for CBD, thereby facilitating dissolution and stability, and the particular reasons still required more profound experimental research and analysis.

In combination with the results of the dissolution test, the determination of droplet size, and PDI, calcium silicate was chosen as the carrier for spray drying in accordance with the ratio of 1:1. PVP VA64 (3% of the amount of L-SNEDDS) was added as PI.

#### 3.3.5. Scanning Particle Size of CBD-Loaded S-SNEDDS-SD Powders

Particle size is an important physical property of solid powders. The particle size result of the carrier of calcium silicate was D_10_ 1.40 ± 0.02 μm, D_50_ 5.44 ± 0.12 μm, D_90_ 17.87 ± 0.12 μm, while the particle size data of S-SNEDDS-F13 (SD powders) were D_10_ 2.58 ± 0.19 μm, D_50_ 6.72 ± 0.32 μm, D_90_ 20.87 ± 0.51 μm.

Compared with the calcium silicate carrier, the particle size of the CBD-loaded formulation did not have a large-scale change. This indicated that the spray drying approach was rather ideal, being capable of realizing the loading of the drug without noticeably altering the fundamental features of the carrier, which was beneficial for maintaining the homogeneity and stability of the formulation.

### 3.4. Preparation and Characterization of Laboratory-Scale S-SNEDDS Powders

#### 3.4.1. Preparation of Laboratory-Scale S-SNEDDS Powders

Via the above investigations, the anhydrous dibasic calcium phosphate (Fujicalin SG) was selected as the solid adsorption carrier (with a 2:1 *w*/*w* ratio of the carrier to L-SNEDDS). In contrast, calcium silicate and PVP VA64 were regarded as the optimal spray drying carrier (with a 1:1 *w*/*w* ratio of the carrier to L-SNEDDS) and precipitation inhibitor (at 3% of L-SNEDDS). Laboratory-scale S-SNEDDS-F14 and F15 formulations were prepared by means of solid carrier adsorption and spray drying method, which are designed in Table 7.

#### 3.4.2. Scanning Flow Properties of S-SNEDDS Powders

The results of the flow properties are presented in Table 7. S-SNEDDS-F14 (S-SNEDDS-SCA) exhibited good flowability with the Carr’s index of 16.09 ± 1.96% and an angle of repose of 27.03 ± 0.93°. In comparison to S-SNEDDS-SCA, the flow properties of S-SNEDDS-F15 (S-SNEDDS-SD) were slightly worse with the Carr’s index of 22.68 ± 1.52% and an angle of repose of 33.41 ± 1.24°. However, the S-SNEDDS-SD could still meet the fundamental flowability requirements of the formulation.

#### 3.4.3. Drug Release of S-SNEDDS Powders

**Figure 9:**
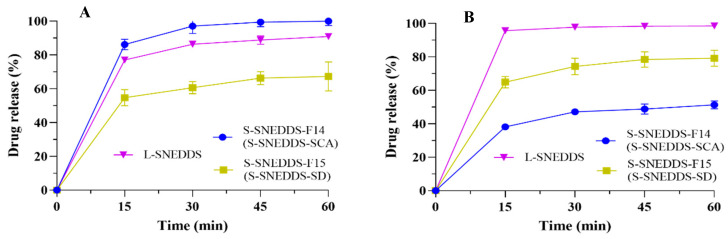
The drug release in (**A**) pH 1.0 and (**B**) pH 6.8 conditions of L-SNEDDS, S-SNEDDS-F14 powder (solid carrier adsorption, SCA), and S-SNEDDS-F15 powder (spray drying, SD). Data are represented as the mean ± S.D. (*n* = 3).

The drug release results of the S-SNEDDS powders are displayed in Figure 9. It was found that the CBD released from L-SNEDDS, S-SNEDDS-F14 (S-SNEDDS-SCA), and F15 (S-SNEDDS-SD) in pH 1.0 conditions was 90.88 ± 1.46%, 99.94 ± 2.58% and 67.25 ± 8.59% in 1 h, respectively. The drug release of the three formulations in pH 6.8 conditions were 98.43 ± 1.18%, 51.32 ± 2.40%, and 79.16 ± 4.73% in 1 h, respectively.

The powders obtained through the carrier adsorption process were consistent with the dissolution end point of L-SNEDDS, and the powders prepared by the spray drying process exhibited a slightly lower dissolution in the pH 1.0 medium. Conversely, in the medium of pH 6.8, the dissolution behaviors of SCA powders were inferior to those of SD powders, and both solid samples had a lower dissolution than the liquid sample. The slower dissolution after the SNEDDS solidification was speculated to be related to the variation in the physical state and interfacial properties of SNEDDS. Solidification means that the nanoemulsion transforms from liquid to solid. In this process, the interaction among emulsion droplets was enhanced, and the interfacial properties changed, possibly forming a more stable configuration, which altered the diffusion path and difficulty for drug molecules, resulting in a slower rate of drug release.

#### 3.4.4. XRD

**Figure 10:**
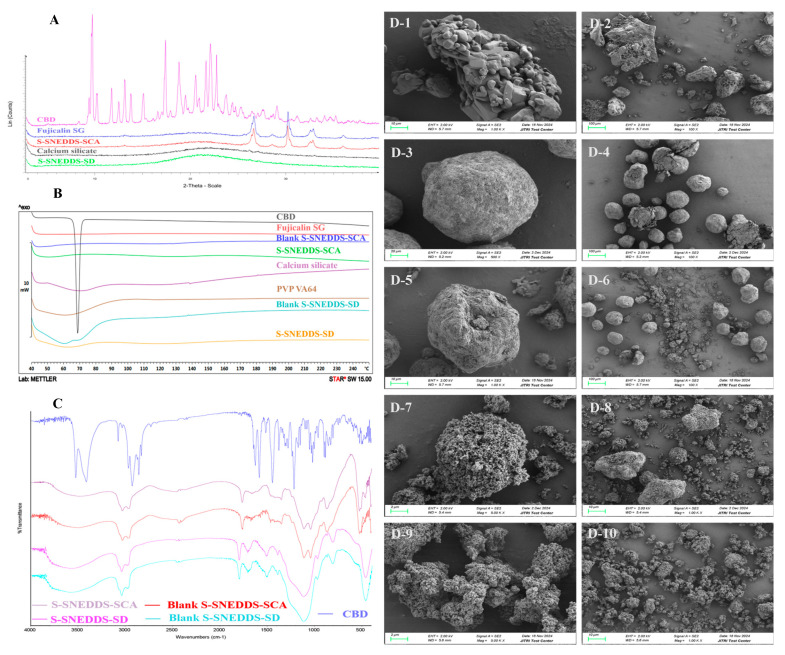
Overlay plots of (**A**) XRD, (**B**) DSC, (**C**) FTIR, and (**D**) SEM. The SEM images at different magnifications and different scale bars: CBD ((**D-1**) ×1000, 10 μm; (**D-2**) ×100, 100 μm), Fujicalin SG ((**D-3**) ×500, 20 μm; (**D-4**) ×100, 100 μm), S-SNEDDS-SCA ((**D-5**) ×1000, 10 μm; (**D-6**) ×100, 100 μm), calcium silicate ((**D-7**) ×5000, 2 μm; (**D-8**) ×1000, 10 μm), and S-SNEDDS-SD ((**D-9**) ×5000, 2 μm; (**D-10**) ×1000, 10 μm).

The results of XRD are depicted in Figure 10A. Over a range of diffraction angles, several representative peaks were exhibited in CBD, indicating crystalline characteristics. The XRD spectra of calcium silicate and CBD-loaded S-SNEDDS-SD showed the absence of any peaks, indicating the amorphous nature of calcium silicate and the final formulation. In contrast, the S-SNEDDS-SCA formulation showed some short peaks, which were also observed in the carrier of Fujicalin SG. S-SNEDDS-F14 (S-SNEDDS-SCA) and F15 (S-SNEDDS-SD) formulations did not display any crystalline characteristic peaks of CBD, suggesting that CBD was molecularly dissolved within the solid SNEDDS or in an amorphous state.

#### 3.4.5. DSC

The DSC analysis results verified that pure CBD exhibited a distinct endothermic peak at 68.24 °C (Figure 10B), confirming its crystallinity. In contrast, pure carriers (Fujicalin SG, calcium silicate, and PVP VA64), blank formulations (blank S-SNEDDS-SCA, blank S-SNEDDS-SD), and CBD-loaded formulations (S-SNEDDS-SCA, S-SNEDDS-SD) demonstrated a complete disappearance of the endothermic peak of CBD within the same temperature range. This finding could reasonably be construed as transforming from a crystalline state to an amorphous state in the CBD-loaded S-SNEDDS.

#### 3.4.6. FTIR

Figure 10C shows the FTIR spectra of pure CBD, blank S-SNEDDSs (blank S-SNEDDS-SCA, blank S-SNEDDS-SD), and CBD-loaded S-SNEDDSs (S-SNEDDS-SCA, S-SNEDDS-SD). The FTIR spectrum of pure CBD showed remarkable molecular vibrations ranging from 3409 cm^−1^ to 3520 cm^−1^, which corresponded to the O-H (phenolic) stretching vibrations, approximately 1581 cm^−1^ signified C=C stretching (aromatic), and the C-O stretching vibrations were at around 1214 cm^−1^. No strong peaks of pure CBD were detected in the FTIR spectra of the SNEDDS formulations. The FTIR spectra of the CBD-loaded S-SNEDDSs were similar to those of blank S-SNEDDS formulations. These results indicated that CBD was completely adsorbed into the solid carriers.

#### 3.4.7. SEM

The scanning electron microscope images of CBD, Fujicalin SG, S-SNEDDS-SCA, calcium silicate, and S-SNEDDS-SD at different magnifications are shown in Figure 10D.

Pure CBD (Figure 10(D-1,D-2)) was oblong or block crystals. The carrier of Fujicalin SG (Figure 10(D-3,D-4)) had a sphere-like shape. The SEM images of S-SNEDDS-SCA (Figure 10(D-5,D-6)) presented the spherical particles, which were similar to the carrier of Fujicalin SG. However, S-SNEDDS-SCA presented a state of twisted, dented, and marginally non-uniform appearance, which would further promote the rapid permeation of water and thereby enable prompt dispersibility within the gastrointestinal environment [50]. The carrier of calcium silicate (Figure 10(D-7,D-8)) and S-SNEDDS-SD formulation (Figure 10(D-9,D-10)) had porous irregular-shaped particles.

The SNEDDS-SCA and S-SNEDDS-SD formulations maintained the shapes of the carriers of Fujicalin SG and calcium silicate, respectively. Meanwhile, no CBD particles were found in the solid S-SNEDDS formulations, indicating that the SNEDDS globules solidified together with the carriers. The obtained results were consistent with XRD and DSC data, which confirmed the existence of drugs in the amorphous state [51].

### 3.5. Preparation and Characterization of S-SNEDDS Unit Dose Formulations

#### 3.5.1. Preparation of S-SNEDDS Unit Dose Formulations

Based on the study on the S-SNEDDS powders, the S-SNEDDS unit dose formulations of capsules and tablets are prepared as shown in Table 8. Here, two processes of SCA and SD were used to transform L-SNEDDS into powders. The S-SNEDDS powders were added to MCC, croscarmellose sodium, and magnesium stearate and mixed evenly. Unit dose formulations such as tablets and capsules were further developed.

#### 3.5.2. In Vitro Drug Release Profile and Nanoemulsion Study

**Figure 11:**
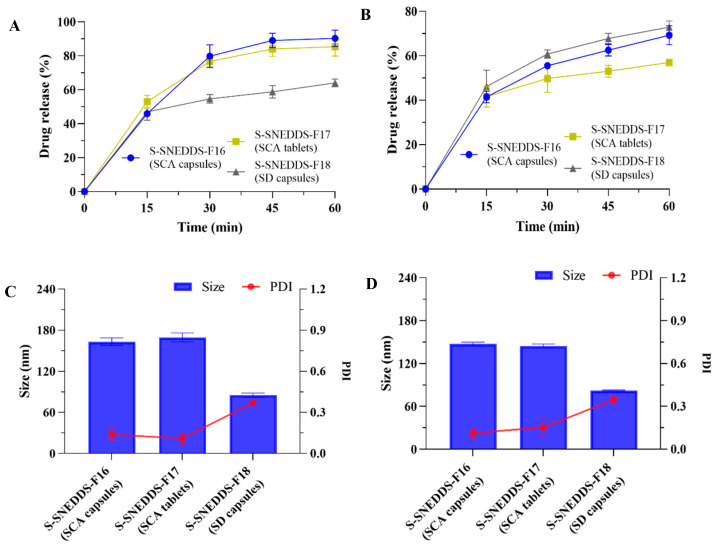
Characterization of CBD-loaded S-SNEDDS unit dose formulations. The dissolution profile in (**A**) pH 1.0 and (**B**) pH 6.8 conditions. The droplet size and PDI value in (**C**) pH 1.0 and (**D**) pH 6.8 conditions (mean ± S.D., *n* = 3).

The drug release results of the S-SNEDDS capsules and tablets are observed in Figure 11. The CBD release from S-SNEDDS-F16 (SCA capsules), F17 (SCA tablets), and F18 (SD capsules) in pH 1.0 conditions was found to be 90.26 ± 4.84%, 85.38 ± 5.54% and 64.02 ± 2.23% in 1 h, respectively. The drug release of the three formulations in pH 6.8 conditions were 69.26 ± 4.27%, 56.94 ± 1.46%, and 72.89 ± 2.76% in 1 h, respectively.

The S-SNEDDS-F16 (SCA capsules) exhibited superior dissolution in the pH 1.0 medium as compared to other formulations. Inversely, in the medium of pH 6.8, the dissolution behaviors of F18 (SD capsules) were higher than those of the SCA formulations. The dissolution behavior trends of unit dose samples in pH 1.0 and 6.8 conditions were consistent with those of powder samples.

In the medium of pH 1.0 and pH 6.8, the droplet size after the solidified preparation disintegration was less than 200 nm. Among them, the PDI value of SD capsules was slightly larger, but still less than 0.4. The solidified formulations exhibited the characteristics of a nanoemulsion after disintegrating in the dissolution medium.

To compare the storage stability of unit dose formulations prepared by two different techniques, we selected the S-SNEDDS-F16 (SCA capsules) and the F18 (SD capsules), both being of the capsule dosage form, for the stability investigation.

#### 3.5.3. Stability Study

S-SNEDDS-F16 (SCA capsules) and S-SNEDDS-F18 (SD capsules) formulations were packaged with double aluminum, and the stability of CBD-loaded S-SNEDDS formulations at accelerated conditions (40 ± 2 °C/RH75 ± 5%) was investigated by evaluating the drug release, XRD, and DSC for 2 months.

**Figure 12:**
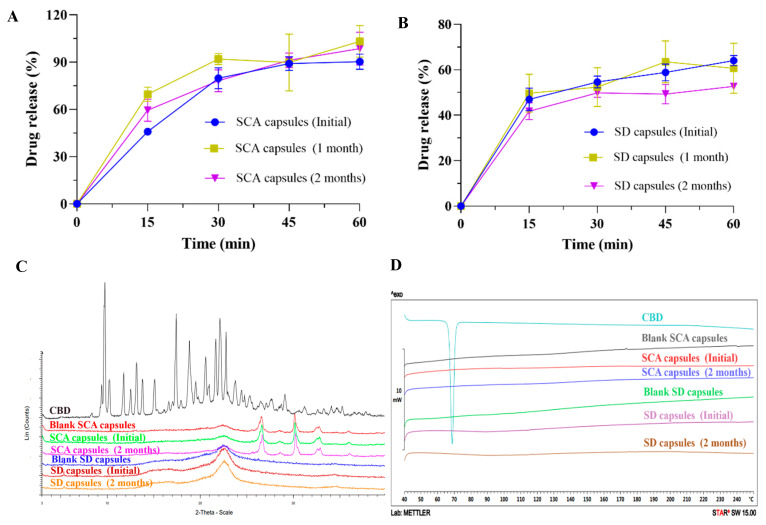
Characterization of CBD-loaded S-SNEDDS capsules at accelerated conditions. The drug release profiles of (**A**) S-SNEDDS-SCA capsules and (**B**) S-SNEDDS-SD capsules in pH 1.0 conditions (mean ± S.D., *n* = 3). (**C**) XRD and (**D**) DSC of the S-SNEDDS formulations (pure CBD and blank capsules formulations were provided as controls).

The drug release profiles are presented in Figure 12A,B. During the period of accelerated stability, the dissolution behaviors were basically consistent with the initial formulations. The XRD and DSC results (Figure 12C,D) demonstrated that the S-SNEDDS formulations exhibited good stability of amorphous state for two months under accelerated conditions, which lacked the crystalline characteristics of CBD, and was consistent with the solid-state characterization of the blank formulations.

### 3.6. In Vivo Pharmacokinetic Study

**Figure 13:**
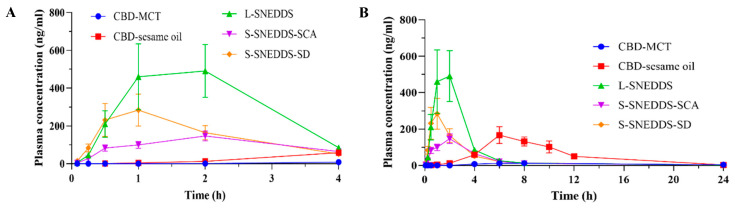
Mean plasma concentration time profile for CBD administered in the SNEDDS formulations in comparison to oil-based formulations (**A**) during the first 4 h and (**B**) during the 24 h following a single oral dose of CBD at 15 mg/kg. *n* = 4 (CBD-sesame oil group), *n* = 3 (the other four groups), error bar = SEM of the mean.

The pharmacokinetics profile of CBD in SNEDDS formulations (L-SNEDDS, S-SNEDDS-SCA, and S-SNEDDS-SD) was compared with that of two oil-based formulations (CBD-MCT and CBD-Sesame oil, as the control) in Figure 13 and Table 9. The Cmax for L-SNEDDS, S-SNEDDS-SCA, S-SNEDDS-SD, CBD-MCT, and CBD-sesame oil was determined to be 499.5 ± 144.6 (L-SNEDDS, highest), 146.7 ± 26.0, 283.7 ± 84.7, 13.4 ± 4.2 (CBD-MCT, lowest), and 186.2 ± 31.4 ng/mL, respectively. The Cmax values showed significant differences among the three S-SNEDDS formulations and CBD-MCT (*p* < 0.05). The Tmax value for CBD-MCT administration (6.7 ± 0.7 h) was similar to that of CBD-sesame oil (6.5 ± 0.5 h), while Tmax for L-SNEDDS, S-SNEDDS-SCA, and S-SNEDDS-SD administration (1.3 ± 0.3, 2.0 ± 0.0, 1.0 ± 0.0 h) was shorter compared with the oil-based formulations. There were significant differences in Tmax values between the three S-SNEDDS and oil-based formulations (*p* < 0.05). Figure 13 suggests that CBD was absorbed rapidly (Tmax within 2 h) following the administration in the SNEDDS, which is rationally attributed to the augmentation of the surface absorption area offered by the nanoemulsification process in the gastrointestinal tract. While the oil-based formulations exhibited a delayed absorption of CBD, lasting until 6~7 h, the SNEDDS formulations led to a rapid absorption rate and a relatively high Cmax.

Besides anti-epilepsy, CBD possesses other abundant biological activities, which include anti-anxiety, sedative, and hypnotic pharmacological activities. A higher Cmax/shorter Tmax is beneficial for rapidly relieving anxiety or improving sleep quality.

As presented in Table 9, the AUC_0–4 h_ value of CBD from SNEDDS formulations was significantly greater than that of oil-based formulations during the first 4 h. Additionally, the AUC value of CBD for SNEDDS formulations was also contrasted with oil-based formulations from 0 to 24 h. Between 0 and 24 h, the AUC value for L-SNEDDS, S-SNEDDS-SCA, S-SNEDDS-SD, CBD-MCT and CBD-sesame oil was 1504.6 ± 427.7 (L-SNEDDS, highest), 624.1 ± 77.0, 808.0 ± 219.4, 109.5 ± 63.3 (CBD-MCT, lowest), 1304.5 ± 191.1 ng/mL·h, respectively. The systemic exposure to CBD following the administration of L-SNEDDS, S-SNEDDS-SD, and S-SNEDDS-SCA formulations increased by 12.7-, 6.4-, and 4.7-fold, respectively, compared with the CBD-MCT administration (*p* < 0.05). However, the ratio of the AUC_0–24 h_ value of CBD relative to CBD-sesame oil was 1.2-, 0.6-, and 0.5-fold for L-SNEDDS, S-SNEDDS-SD, and S-SNEDDS-SCA formulations, respectively. There were no significant increases in the AUC_0–24 h_ values between CBD-sesame oil and the three S-SNEDDS formulations, which had already been reported in the previous works of literature [22,52].

In this study, the Cmax and systemic exposure in vivo after the oral administration of CBD-MCT oil are inconsistent with the previous research [53]. The previous research into CBD formulations in MCT oil has revealed much higher plasma concentrations (16-fold greater). It is speculated that the reason for this inconsistency is related to the prescriptions and food effect, and the animals’ variations and administration dosage also exert influence. The observed Cmax disparity between the current CBD-MCT formulation (pure MCT excipient) and prior studies (commercial CBD oil with likely ethanol/solubilizer) might stem from additive-enhanced solubility and permeability in the latter, critically influencing the systemic exposure. The feeding time of the animals was different in the two studies; the rats were fed 6 h after the oral administration (this study) and 2 h after (previous research). These factors all influenced the Cmax and in vivo exposure.

As a highly lipophilic drug, CBD has low solubility, incomplete oral absorption, obvious first-pass effect in the liver. These factors lead to significant differences in oral bioavailability among the individuals [54]. Experimental design, formulation, and sample size also affect the pharmacokinetic results. The food effect further aggravates this difference. The CBD oral solution (Epidiolex) approved by the US FDA discloses that coadministration of Epidiolex with a high-fat/high-calorie meal increased the Cmax by 5-fold, AUC by 4-fold, and reduced the total variability, compared with the fasted state in healthy volunteers [5].

Currently, there are relatively few studies on the food effect of the SNEDDS formulations. The presence of food may affect the dispersion and emulsification processes of the SNEDDS formulations. Food may also play a role in promoting the absorption by delaying gastric emptying and prolonging the residence time of the SNEDDS in the gastrointestinal tract.

In this study, the variability of the PK parameters is slightly greater, especially in the SNEDDS formulations and CBD-MCT. It is speculated that this may be related to the individual differences in rats and the relatively small animal sample size. It is considered that increasing the sample size could reduce the difference in PK data, and employing large animals (such as beagles) could minimize the adverse impact of the small gastric juice volume on the self-emulsifying performance.

It could be observed from the PK data that the CBD-sesame oil formulation was capable of achieving sustained release in vivo, and the L-SNEDDS had the largest in vivo exposure. Solid SNEDDS formulations exhibit good flow properties, and the solid powders possess high scalability to develop more solid dosage forms, whereas the advantage of enhancing bioavailability was not obvious, and the systemic exposure of S-SNEDDS formulations was relatively low. As in the aforementioned analysis, it was speculated that the solid carrier affected the release of CBD in the S-SNEDDS formulations, leading to the relatively low in vivo exposure. The studies on the carrier, formulation, and technology of the S-SNEDDSs are expected to improve in vivo exposure.

The enhanced pharmacokinetic profile of the SNEDDS formulations, which is characterized by a higher Cmax and an accelerated Tmax, enables the rapid attainment of therapeutic drug concentrations. This is a crucial advantage for CBD in time-sensitive therapeutic applications such as the relief of anxiety and improvement of sleep quality. The rapid systemic exposure not only sustains prompt pharmacological action but also may enhance treatment efficacy by aligning peak drug levels with the onset of symptom-driven demands.

## 4. Conclusions

In this research, a novel SNEDDS of CBD was developed with the excipients of oil (MCT, 10%, *w*/*w*), surfactants (Labrasol, 36%, *w*/*w*; Tween 80, 36%, *w*/*w*), and co-surfactant (Transcutol, 18%, *w*/*w*). The preferred SNEDDS exhibited remarkable nano-formulation characteristics, excellent in vitro drug release, and good thermodynamic stability. The liquid SNEDDS formulations were transformed into the solid SNEDDS powders through solid carrier adsorption (carrier: Fujicalin SG) and spray drying (carrier: calcium silicate, precipitation inhibitor: PVP VA64) technologies. The two S-SNEDDS powders possessed good flow properties, and were further converted into unit dose formulations (capsules and tablets), displaying good in vitro drug release profile and self-emulsification performance. Accelerated stability studies indicated that S-SNEDDS capsules demonstrated excellent storage stability. Meanwhile, compared with the two oil-based formulations (CBD-sesame oil (similar to Epidiolex^®^) and CBD-MCT), oral administration of L-SNEDDS and spray drying S-SNEDDS formulations resulted in faster absorption of CBD (Tmax: 6~8 h (CBD oils), 1~2 h (SNEDDS formulations); *p* < 0.05 compared with two controls) and higher blood concentrations (*p* < 0.05 compared with CBD-MCT), which were essential for treatment neurological diseases.

In summary, SNEDDS is a potential oral delivery system to improve the solubility and oral absorption of CBD. Solid SNEDDS offers scalability and patient compliance, and it is capable of being modified into more convenient solid dosage forms, thereby accelerating the development of CBD-related solid drugs and nutraceuticals. Additionally, SNEDDS can be extended to the development of other drugs with poor water solubility.

## Figures and Tables

**Table 1:** Nanoemulsion regions of the pseudo-ternary phase diagrams in different formulations with different ratios of mixed surfactants.

Oils	Surfactants (Smix, Larbrasol: Tween 80)	Region
**MCT**	only Labrasol	12.5%
	only Tween 80	6.8%
	3:1	8.9%
	2:1	16.5%
	**1:1**	**20.0%**
	1:2	13.9%
	1:3	8.1%

The ratio of surfactant and co-surfactant (Transcutol) was 4:1. The bold part was the final chosen prescription.

**Table 2:** The prescription design of CBD-loaded SNEDDS and controls.

Samples	Composition (*w*/*w*)				
	MCT	Sesame Oil	Labrasol	Tween 80	Transcutol
**L-SNEDDS-F01**	**10%**	**/**	**36%**	**36%**	**18%**
L-SNEDDS-F02	15%	/	34%	34%	17%
L-SNEDDS-F03	20%	/	32%	32%	16%
L-SNEDDS-F04	/	10%	36%	36%	18%
Control groups (oil-based formulations, *w*/*w*)					
CBD-MCT	100%MCT				
CBD-sesame oil	92.03% sesame oil, 7.9% dehydrated alcohol, 0.05% sucralose, and 0.02% Strawberry flavor				

The concentration of CBD-loaded formulation was 30 mg CBD/1 g formulations. The bold part was the final chosen prescription.

**Table 3:** The oil adsorption capacity of different solid carriers (*n* = 3).

Type of Carriers	Chemical Name	Company	Oil Adsorption Capacity (g Oils/1 g Carrier)
SYLOID 244FP	silicon dioxide	Grace	1.80
SYLOID XDP 3050		Grace	2.05
SYLOID XDP 3150		Grace	2.21
Neusilin UFL2	magnesium aluminum metasilicate	Fuji	2.68
Neusilin US2		Fuji	2.52
Fujicalin SG	anhydrous dibasic calcium phosphate	Fuji	1.55
DI-CAFOS A150		Budenheim	1.40

**Table 4:** Study of the prescriptions, flow properties, and compressibility of S-SNEDDS.

Samples	S-SNEDDS-F8	S-SNEDDS-F9	S-SNEDDS-F10
carrier species	SYLOID XDP 3150	Neusilin UFL2	Fujicalin SG
ingredients	Composition (%)		
solid carrier	25%	25%	62%
L-SNEDDS	25%	25%	31%
MCC	43%	43%	/
HPC	2%	2%	2%
croscarmellosesodium	4%	4%	4%
magnesium stearate	1%	1%	1%
Flow properties of powders (solid carrier to L-SNEDDS)			
Carr’s index	11.18 ± 1.05%	14.75 ± 0.76%	15.21 ± 1.05%
the angle of repose	25.78 ± 0.55°	27.63 ± 1.41°	29.74 ± 0.88°
Compressibility of tablets			
record of compression	stress crack of tablets	smooth surface with no splinters	smooth surface with no splinters

**Table 5:** Study on the solubility of PI.

Name	PVP K25	PVP K30	PVP VA64	Poloxamer 407
ratio of addition	3%	3%	3%	3%
solubility observation	slow dissolution, and there was a small amount of undissolved solution at the bottom		completely dissolved	always undissolved

**Table 7:** Study on the prescriptions, and flow properties of laboratory-scale S-SNEDDS powders.

Samples	S-SNEDDS-F14	S-SNEDDS-F15
techniques	solid carrier adsorption (SCA)	spray drying (SD)
carrier species	Fujicalin SG	calcium silicate
PI	/	PVP VA64
prescription	Fujicalin SG:L-SNEDDS (200 g:100 g)	calcium silicate:L-SNEDDS:PVP VA64 (100 g:100 g:3 g)
Flow properties of S-SNEDDS powders		
Carr’s index	16.09 ± 1.96%	22.68 ± 1.52%
the angle of repose	27.03 ± 0.93°	33.41 ± 1.24°

**Table 8:** Study on S-SNEDDS unit dose formulations.

Sample	S-SNEDDS-F16	S-SNEDDS-F17	S-SNEDDS-F18
techniques	solid carrier adsorption (SCA)	solid carrier adsorption (SCA)	Spray drying (SD)
carrier species	Fujicalin SG	Fujicalin SG	calcium silicate
dosage form	capsules	tablets	capsules
ingredients	Composition (%)		
L-SNEDDS	22.5%	22.5%	31.6%
solid carrier	45.0%	45.0%	31.6%
PVP VA64	/	/	0.1%
MCC	27.5%	27.5%	31.6%
croscarmellose sodium	4.0%	4.0%	4.0%
magnesium stearate	1.0%	1.0%	1.1%
weight of tablets or capsule contents	196 ± 9.8 mg	196 ± 9.8 mg	158 ± 7.9 mg

**Table 9:** Pharmacokinetic parameters obtained following the oral administration of CBD-loaded SNEDDS and oil-based formulations in rats (*n* = 3–4). All values are presented as mean ± SEM.

Group	Cmax (ng/mL)	AUC_0–4 h_ (ng/mL·h)	AUC_0–24 h_ (ng/mL·h)	AUC_0–∞_ (ng/mL·h)	Tmax (h)	T_1/2_ (h)
L-SNEDDS	499.5 ± 144.6 *	1255.4 ± 368.4 *#	1504.6 ± 427.7 *	1534.3 ± 438.9	1.3 ± 0.3 **##	4.8 ± 1.8
S-SNEDDS-SD	283.7 ± 84.7 *	616.2 ± 162.2 *#	808.0 ± 219.4 *	833.8 ± 214.7	1.0 ± 0.0 **###	4.5 ± 1.2
S-SNEDDS-SCA	146.7 ± 26.0 **	396.2 ± 62.3 *##	624.1 ± 77.0 **#	642.5 ± 77.4	2.0 ± 0.0 **##	5.9 ± 0.2
CBD-MCT	13.4 ± 4.2	9.4 ± 3.7	109.5 ± 63.3	/	6.7 ± 0.7	/
CBD-sesame oil	186.2 ± 31.4	80.4 ± 11.6	1304.5 ± 191.1	1317.5 ± 192.0	6.5 ± 0.5	3.0 ± 0.3

/ The AUC_0–∞_ and T_1/2_ of CBD-MCT were unable to be acquired on account of an inadequate quantity of data points within the elimination stage. Statistical significance between CBD-sesame oil and CBD-MCT was not considered. Statistical difference in Cmax, AUC_0–t_, Tmax were indicated as follows: * *p* < 0.05, ** *p* < 0.01, compared to CBD-MCT. # *p* < 0.05, ## *p* < 0.01, ### *p* < 0.001, compared to CBD-sesame oil.

## Data Availability

All data are contained within the article.
